# Special Issue on “Function of Polymers in Encapsulation Process”

**DOI:** 10.3390/polym14061178

**Published:** 2022-03-15

**Authors:** M. Ali Aboudzadeh, Shaghayegh Hamzehlou

**Affiliations:** 1 CNRS, Institut des Sciences Analytiques et de Physico-Chimie pour l’Environnement et les Matériaux, University Pau & Pays Adour, E2S UPPA, IPREM, UMR5254, 64000 Pau, France; m.aboudzadeh-barihi@univ-pau.fr; 2 Polymat and Kimika Aplikatua Saila, Kimika Fakultatea, University of the Basque Country UPV-EHU, Joxe Mari Korta Zentroa, Tolosa Hiribidea 72, 20018 Donostia-San Sebastian, Spain

Encapsulation technology comprises enclosing active agents (core materials) within a homogeneous/heterogeneous matrix (wall material) at the micro/nano scale. In the last few years encapsulation has gained a lot of interest. Using this process, a physical barrier is developed between the inner substance and the environment which on one hand prevents its degradation and facilitates its handling and transportation and on the other hand allows the controlled release of the core material in a certain ambiance [1]. Polymers may be used to trap the material of interest inside the micro/nano-capsules. Such encapsulated systems have many applications in the fields of the food industry, drug delivery, agriculture, cosmetics, coatings, adhesives and so forth. Various biopolymers, such as alginate, chitosan, carrageenan, gums, gelatin, whey protein or starch, act as a barrier against external conditions. Encapsulation in biodegradable polymers can also enhance the permeability and stability of the active agent and thus its bioavailability. Choosing the right polymer is very important in this process due to its impact on target delivery and controlled release, and therefore, on the bioavailability of active agents. It should have the necessary properties, such as being non-reactive with the active agent, flexibility, stability, strength, and impermeability. If the active agent has application in the food industry, the used polymer should be “generally recognized as safe” (GRAS), biodegradable, and capable of preserving the encapsulated material from the atmosphere [2].

There are a number of chemical, physical or mechanical processes available for encapsulation such as emulsion-solvent evaporation/extraction methods, coacervation-phase separation, spray drying, interfacial and in situ polymerization. The choice of a particular technique depends on the attributes of the polymer and the active agent. There are still many aspects to be developed in this field, which offer new challenges and breakthrough opportunities. The main objective of this interdisciplinary Special Issue is to bring together, at an international level, a high-quality collection of reviews and original research articles dealing with the importance of natural or synthetic polymers in encapsulation processes and their applications. A deep understanding and relevant theoretical calculations for exploring the functions of the materials (involved in the formulations) have also been obtained by fundamental investigations. The purpose of this editorial is to provide a brief introduction for each published or accepted paper and highlight their major findings and discoveries. In the following, we review all the articles in our Special Issue, and we believe this editorial will interest the broadest possible section of readership.

One of the crucial characteristics of drug delivery systems in biomedicine is to impart the sustained release of an encapsulated drug. Thus, the sustained release enhances the accumulation of the drug at the target site while improving its bioavailability. Aboudzadeh et al. in three studies (published in this Special Issue) explored the solubility, permeability, oral bioavailability, and therapeutic effectiveness of different drugs through their encapsulation with polymers, intending to achieve an enhanced drug release. In the first study, lignin the second most abundant biopolymer on earth (after cellulose) was used to encapsulate doxorubicin (DOX) in oil-in-water (O/W) microemulsions to enhance the bioavailability of DOX. The authors demonstrated that encapsulation and slow release of DOX enhanced the cytotoxic efficacy of this anthracycline agent against cancer cells but did not improve its safety towards normal human cells [3]. In another report, Aboudzadeh et al. designed a delivery system based on ethylcellulose (a biobased polymer) nanosponges that could improve the oral bioavailability of Olmesartan medoxomil, one of the prominent antihypertensive drugs. It was shown that the optimized nanosponge drug carrier presented a better-sustained release with improved antihypertensive efficacy and antitumor activity against A549 lung cancer cell lines [4]. In the last study, three hydrophilic polymers, namely Kolliphor^®^ P188, Kollidon^®^ 30, and Kollidon^®^-VA 64 (obtained from BASF Co., Ludwigshafen, Germany) were employed as drug carriers through solid dispersion (SD) approach to encapsulate Sildenafil citrate, a frequently used medication (under the brand name Viagra) for the treatment of erectile dysfunction (ED). The authors concluded that the optimized SD system exhibited superior activity and was a promising strategy for improving solubility, dissolution rate, and aphrodisiac effects on male rats [5].

Another poorly water-soluble drug, artemisinin, was formulated by Natesan et al. into nanomicelles using polyvinylpyrrolidone (PVP K90) and a polymeric surfactant (Poloxamer 407). Artemisinin-loaded nanomicelles presented a notable anti-angiogenic activity compared to artemisinin suspension. The authors proposed this formulation as a potential treatment for age-related macular degeneration (AMD) [6]. In another report published by Sylwia Łukasiewicz, clozapine the second-generation antipsychotic drug, was encapsulated within polymeric nanocapsules using an anionic surfactant (sodium docusate) and bio-compatible polyelectrolytes such as poly-_L_-glutamic acid (PGA) and poly-_L_-lysine (PLL). The outer layer of the carrier was grafted by polyethylene glycol (PEG). According to the author, this approach may facilitate the availability and safety of the drug, enhance the selectivity of its performance, and subsequently increase the efficiency of schizophrenia therapy [7].

Nowadays, one of the main issues in microencapsulation processes is obtaining a stable product during storage with consistent encapsulation and release efficacy. The latter requirement is relatively difficult to achieve because of the sensitivity of common materials (used for matrix formulation) to processing and environmental factors that could lead to poor encapsulation efficiency, short microcapsules shelf-life and uncontrolled release kinetics. In this context, natural polymer-based carriers (with core–shell structure) have presented multifunctional capabilities and enabled the simultaneous encapsulation of active agents of different physical/chemical properties for their targeted delivery and sustained release. Design of micro- and nano-metric core–shell capsules and porous spheres made of natural polymers (e.g., such as polysaccharides and polypeptides), their function-specific aspects, mechanisms of formation, challenges, and future perspectives are discussed in a review paper written by Gedanken et al. published in this issue [8].

Another interesting type of polymers are hydrogels, which are utilized in many fields and more particularly in the biomedical field where they can function as drug protectors, targetable carriers for bioactive agents, etc. Jean-François Gohy et al. contributed in this issue by developing a new class of redox-responsive hydrogels by combining 2,2,6,6-tetramethyl-1-piperidinyloxy (TEMPO) stable nitroxide radicals and oligoethylene glycol methyl ether methacrylate (OEGMA). The authors showed these polymeric networks can be successfully used either for the electrochemically triggered release of aspirin or as catalysts for the oxidation of primary alcohols into aldehydes [9]. Various chemical and physical stimulants may serve as triggers for drug release. Variations in ionic strength and ionic composition combined with pathological processes may provide triggers to cause a carrier response. Recent developments and novel strategies in designing ion-responsive drug delivery systems including a variety of structures were reviewed by Musiał et al. [10]. Another type of smart material is phase-changing materials (PCMs). Microencapsulation is an applicable procedure to conserve and retain the characteristics of PCMs that are used in thermal energy storage applications. Skurkyte-Papieviene et al., in an interesting study, improved the thermal performance of paraffin PCM microcapsules (consisting of a polymeric melamine-formaldehyde) for textile application by modifying their outer shell [11].

Despite these creative reports in the field of smart polymers, the need to create new polymer lattices has always existed owing to the broad plethora of industrial applications involved. According to this necessity, Cappelletti et al. developed butyl methacrylate-based lattices via free radical polymerization in water by adding (i) methacrylic acid/methyl methacrylate and (ii) methacrylamide, respectively, as an aqueous phase in Pickering emulsions. Both synthesized lattices displayed promising characteristics as protective polymer shells for the controlled delivery of active agents [12].

From an engineering point of view, the continuously increasing demand for cost reduction, together with short-time processes is leading to substantial research developments in new methods for loading bioactive agents. The coating containing bioactive agents is one of the most successful approaches for engineering material surfaces while keeping bulk properties. An interesting article of this issue reports direct deposition of poly(acrylic acid) coatings containing various agents (e.g., dyes, fluorescent molecules) through aerosol-assisted open-air plasma method. In this work, Mantovani et al. employed this one-step process potentially to encapsulate a tunable quantity of any sensible water-soluble agent without changing its activity. They presented this approach as quite appealing for designing specific drug-release systems [13].

Finally, the last contribution of this Special Issue focuses specifically on the significance and emerging role of nanotechnology to treat pollutants in water (e.g., 4-NP and morin) and improve its quality. In this study, amphiphilic polyethyleneimine (PEI)-oleic acid micelles-stabilized palladium nanoparticles were synthesized by Wang et al., which effectively catalyzed the reduction of 4-NP to form 4-AP and had high catalytic efficiency against morin. The authors concluded that these nanoparticles have great potential applications in treating various organic contaminants in solution in the future [14].

In view of the above summary and discussion, we believe that the present Special Issue explored the latest research on the function of polymers in encapsulation technology including fundamental theory and experiments together with reviews and articles. More efficient designs and preparation processes, as well as further understandings of the interfacial chemistry of encapsulated materials within the polymeric systems, are needed.
